# The Formula of Grangeat for Tensor Fields of Arbitrary Order in *n* Dimensions

**DOI:** 10.1155/2007/12839

**Published:** 2007-03-08

**Authors:** T. Schuster

**Affiliations:** Department of Mechanical Engineering, Helmut Schmidt University, P.O. Box 700822, 22008 Hamburg, Germany

## Abstract

The cone beam transform of a tensor field of order *m* in *n* ≥ 2 dimensions is considered. We prove that the image of a tensor field under this transform is related to a derivative of the *n*-dimensional Radon transform applied to a projection of the tensor field. Actually the relation we show reduces for *m* = 0 and *n* = 3 to the well-known formula of Grangeat. In that sense, the paper contains a generalization of Grangeat's formula to arbitrary tensor fields in any dimension. We further briefly explain the importance of that formula for the problem of tensor field tomography. Unfortunately, for *m* > 0, an inversion method cannot be derived immediately. Thus, we point out the possibility to calculate reconstruction kernels for the cone beam transform using Grangeat's formula.

## 1. INTRODUCTION

The cone beam transform for a symmetric covariant tensor field **f** of order *m* reads as

(1) $$\text{Df}\left(a,\omega \right)=\int_{0}^{\infty }\langle \text{f}\left(a+t\omega \right),\omega^{m}\rangle \text{d}t$$,

where *a* is the source of an X-ray, *ω* ∈ *S* ^*n*−1^ is a direction, and *ω^m^* denotes the *m*-fold tensor product *ω^m^ = ω ⊗ ⋯ ⊗ ω*. If *m* = 0, this is the classical X-ray transform of functions which represents the mathematical model for the cone beam geometry in computerized tomography. For *m* = 1, the operator **D** is the longitudinal X-ray transform of vector fields. A lot of numerical algorithms have been developed in recent years to solve the inverse problem **D** **f** = *g* in case *m* = 0 and *m* = 1; see, for example, Louis [1], Katsevich [2], Schuster [3], Derevtsov and Kashina [4], Sparr et al. [5] among others. But also for tensor fields of order *m* > 1, this transform is of interest in various applications such as photoelasticity and plasma physics. Solution approaches for the tensor tomography problem are found in Derevtsov [6], and Kazantsev and Bukhgeim [7]. A further important transform in computerized tomography is given by the Radon transform

(2) $$\text{R}f\left(s,\omega \right)=\int_{\omega^{⊥}}f\left(s\omega +y\right)\text{d}y,\;\;\;\;s\in \mathbb{R}$$,

which maps a scalar function to its integrals over hyperplanes.

An important connection between **D** and **R** is given by the formula of Grangeat:

(3) $$\frac{\partial }{\partial s}\text{R}f\left(\omega ,s=\langle a,\omega \rangle \right)=-\int_{S^{2}}\text{D}f\left(a,\theta \right)\delta^{\prime }\left(\langle \theta ,\omega \rangle \right)\text{d}\theta$$,

which is valid for differentiable scalar fields *f* with compact support; see Grangeat [8]. In this paper, we prove a generalization of Grangeat's formula to arbitrary tensor fields. More explicitly, we show that

(4) $$\frac{\partial^{\left(n-2\right)}}{\partial s^{\left(n-2\right)}}\text{R}f_{a}\left(\omega ,s=\langle a,\omega \rangle \right)={\left(-1\right)}^{\left(n-2\right)}\int_{S^{n-1}}\text{Df}\left(a,\theta \right)\delta^{\left(n-2\right)}\left(\langle \theta ,\omega \rangle \right)\text{d}\theta$$,

where *δ* is Dirac's delta distribution and *f_a_* are projections of the tensor field **f**.

In Section 2, we prove that **D** is a bounded linear mapping between suitable *L^2^*-spaces and give a representation for its adjoint D*. In Section 3, we prove formula (4) using a duality argument for **D** and **R**. We finish this paper by pointing out the importance of this result for research in the area of tensor field tomography.

## 2. THE CONE BEAM TRANSFORM OF TENSOR FIELDS

We consider the Euclidean space ℝ*^n^*. A covariant tensor of order *m* in ℝ*^n^* is given by

(5) $$\text{f}=f_{i_{1}\cdots i_{m}}dx^{i_{1}}⊗\cdots ⊗dx^{i_{m}},\;\;\;\;x\in {\mathbb{R}}^{n}$$,

where $f_{i_{1}\cdots i_{m}}$ ∈ ℝ, 1 ≤ *i_j_* ≤ *n* for *j* = 1, … , *m* and *dx^i^*, *i* = 1, … , *n*, is the basis of covectors in (ℝ*^n^*)*,

(6) $$dx^{i}\left(v\right)=v_{i},\;\;\;\;i=1,\ldots ,n,\;\;v\in {\mathbb{R}}^{n}$$.

As in (5), we use Einstein's summation convention throughout the paper, that means we sum up over equal indices. A tensor (5) of order *m* is symmetric if

(7) $$f_{i_{\sigma \left(1\right)}\cdots i_{\sigma \left(m\right)}}=f_{i_{1}\cdots i_{m}}$$,

where *σ* runs over all *m*! permutations of {1, … ,*m*}. The set of all symmetric tensors of order *m* is denoted by *𝒮^m^*. A scalar product on *𝒮^m^* is given by

(8) $$\langle \text{f},\text{g}\rangle =f_{i_{1}\cdots i_{m}}g^{i_{1}\cdots i_{m}},\;\;\;\;\text{f},\text{g}\in {𝒮}^{m}$$,

where $g^{i_{1}\cdots i_{m}}$ are the contravariant components of the tensor **g**. We write $\left\|\text{f}\right\|=\sqrt{\langle \text{f},\text{f}\rangle }$ for the norm on *𝒮^m^*. If *m* = 1, this is the Euclidean norm. A symmetric covariant tensor field of order *m* in ℝ*^n^* maps a point *x* ∈ ℝ*^n^* to an element of *𝒮^m^*,

(9) $$x\mapsto \text{f}\left(x\right)=f_{i_{1}\cdots i_{m}}\left(x\right)dx^{i_{1}}⊗\cdots ⊗dx^{i_{m}},\;\;\;\;x\in {\mathbb{R}}^{n}$$,

where $f_{i_{1}\cdots i_{m}}$ (*x*) ∈ *𝒮^m^* for fixed *x*.

Let further Ω*^n^* = {*x* ∈ ℝ*^n^* : |*x*| < 1} be the open unit ball in ℝ*^n^*. We introduce an inner product for tensor fields defined on Ω*^n^* by

(10) $${\langle \text{f},\text{g}\rangle }_{L^{2}}=\int_{\Omega^{n}}\langle \text{f}\left(x\right),\text{g}\left(x\right)\rangle \text{d}x=\int_{\Omega^{n}}f_{i_{1}\cdots i_{m}}\left(x\right)g^{i_{1}\cdots i_{m}}\left(x\right)\text{d}x,$$

which turns *L* ^2^(Ω*^n^*, 𝒮*^m^*) := {**f** ∈ *𝒮^m^* : ${\left\|\text{f}\right\|}_{L^{2}}={\langle \text{f},\text{f}\rangle }_{L^{2}}^{1/2}$ < ∞} to a Hilbert space. Assume that Γ ⊂ $\left({\mathbb{R}}^{n}\backslash \bar{\Omega^{n}}\right)$ is the path representing the curve of sources of the X-ray beams. Examples for Γ which are used in practice are a circle, two perpendicular circles, or a helix. The cone beam transform of a symmetric tensor field **f** of order *m* is then defined by

(11) $$\begin{array}{c} \text{Df}\left(a,\omega \right)=\int_{0}^{\infty }\langle \text{f}\left(a+t\omega \right),\omega^{m}\rangle \text{d}t\\ =\int_{0}^{\infty }f_{i_{1}\cdots i_{m}}\left(a+t\omega \right)\omega^{i_{1}}\cdots \omega^{i_{m}}\text{d}t, \end{array}$$,

where *ω* ∈ *S* ^*n*−1^ = *∂*Ω*^n^* is the direction and *a* ∈ Γ the source of the beam and *ω^m^ = ω ⊗ ⋯ ⊗ ω* means the *m*-fold tensor product of *ω*. As an arrangement, we extend **f**(*x*) = 0 in ${\mathbb{R}}^{n}\backslash \bar{\Omega^{n}}$. Hence, integrals like (11) are well defined. Finally, we denote **D** *_a_* **f**(*ω*) := **D** **f**(*a, ω*). We note that **D** coincides with the *longitudinal ray transform* in the book of Sharafutdinov [9]. The operators **D** and **D** *_a_* are linear and bounded between *L* ^2^-spaces.

**Theorem 1.**

*Let a* ∈ Γ. *The mappings* **D** *_a_* : *L* ^2^(Ω*^n^*, *𝒮^m^*) → *L* ^2^(*S* ^*n*−1^) *and* **D** : *L* ^2^(Ω*^n^*, *𝒮^m^*) → *L* ^2^(Γ × *S* ^*n*−1^) *are linear and bounded if*

(12) $$\int_{\Gamma }{\left(\left|a\right|-1\right)}^{1-n}\text{d}a<\infty .$$

**Proof.**

For **f** ∈ *L* ^2^(Ω*^n^, 𝒮^m^*) and *a* ∈ Γ, we have

(13) $$\begin{array}{c} \int_{S^{n-1}}{\left|{\text{D}}_{a}\text{f}\left(\omega \right)\right|}^{2}\text{d}\omega =\int_{S^{n-1}}{\left|\int_{0}^{\infty }\langle \text{f}\left(a+t\omega \right),\omega^{m}\rangle \text{d}t\right|}^{2}\text{d}\omega \\ \leq 2\int_{S^{n-1}}\int_{0}^{\infty }{\left\|\text{f}\left(a+t\omega \right)\right\|}^{2}\text{d}t\;\text{d}\omega \\ =2\int_{\Omega^{n}}{\left\|\text{f}\left(x\right)\right\|}^{2}{\left|x-a\right|}^{1-n}\text{d}x\\ \leq 2{\left(\left|a\right|-1\right)}^{1-n}{\left\|\text{f}\right\|}_{L^{2}}^{2}, \end{array}$$

where we used the substitution *x = a* + *tω* and the fact that **f**(*x*) = 0 in ${\mathbb{R}}^{n}\backslash \bar{\Omega^{n}}$. This shows the continuity of **D** *_a_*. The continuity of **D** follows then by using **D** **f**(*a, ω*) = **D** *_a_* **f**(*ω*) and

(14) $${\int_{\Gamma }\int_{S^{n-1}}\left|\text{Df}\left(a,\omega \right)\right|}^{2}\text{d}\omega \;\text{d}a\leq 2{\left\|\text{f}\right\|}_{L^{2}}^{2}\int_{\Gamma }{\left(\left|a\right|-1\right)}^{1-n}\text{d}a.$$

Theorem 1 implies that **D** *_a_* and **D** have bounded adjoints ${\text{D}}_{a}^{*}$ and D*.

**Lemma 1.**

*The adjoints* ${\text{D}}_{a}^{*}$ : *L* ^2^(*S* ^*n*−1^) → *L* ^2^(Ω*^n^, 𝒮^m^*) *and* D* : *L* ^2^(Γ × *S* ^*n*−1^) → *L* ^2^(Ω*^n^, 𝒮^m^*) *have the following representations:*

(15) $${\text{D}}_{a}^{*}g\left(x\right)={\left|x-a\right|}^{1-n-m}g\left(\frac{x-a}{\left|x-a\right|}\right){\left(x-a\right)}^{m}$$,

(16) $${\text{D}}^{*}g\left(x\right)=\int_{\Gamma }\left\{{\left|x-a\right|}^{1-n-m}g\left(\frac{x-a}{\left|x-a\right|}\right){\left(x-a\right)}^{m}\right\}\text{d}a.$$

*In* (15), (16), *the power m again is to be understood as the m-fold tensor product*

(17) $${\left(x-a\right)}^{m}=\left(x-a\right)⊗\cdots ⊗(x-a).$$

**Proof.**

Let **f** ∈ *L* ^2^(Ω*^n^, 𝒮^m^*), *g* ∈ *L* ^2^(*S* ^*n*−1^). Then

(18) $$\begin{array}{c} \int_{S^{n-1}}{\text{D}}_{a}\text{f}\left(\omega \right)g\left(\omega \right)\text{d}\omega =\int_{S^{n-1}}\int_{0}^{\infty }f_{i_{1}\cdots i_{m}}\left(a+t\omega \right)\omega^{i_{1}}\cdots \omega^{i_{m}}g\left(\omega \right)\text{d}t\;\text{d}\omega \\ =\int_{\Omega^{n}}{\left|x-a\right|}^{1-n}f_{i_{1}\cdots i_{m}}\left(x\right)\frac{{\left(x-a\right)}^{i_{1}}\cdots {\left(x-a\right)}^{i_{m}}}{{\left|x-a\right|}^{m}}\times g\left(\frac{x-a}{\left|x-a\right|}\right)\text{d}x\\ ={\langle \text{f},{\text{D}}_{a}^{*}g\rangle }_{L^{2}}. \end{array}$$

Here, again we substituted *x* = *a + tω*. This shows the representation of ${\text{D}}_{a}^{*}$. Equation (16) follows easily from (15) by an integration over Γ.

For *m* = 0, *n* = 3, D* is the backprojection operator in classical 3D cone beam tomography. If *m* = 1, *n* = 3, we obtain the adjoint of the cone beam transform in vector field tomography

(19) $${\text{D}}^{*}g\left(x\right)=\int_{\Gamma }{\left|x-a\right|}^{-3}g\left(a,\frac{x-a}{\left|x-a\right|}\right)\left(x-a\right)\text{d}a.$$

**Remark 1.**

Note that the integrals (12) and (16) are well defined since Γ has a positive distance from $\bar{\Omega^{n}}$.

To prove formula (4), we will also need the adjoint of the Radon transform. The following lemma summarizes basic results of the Radon transform (2) which can be found, for example, in the book of Natterer [10].

**Lemma 2.**

*The transforms* **R** : *L* ^2^(Ω^*n*^) → *L* ^2^([−1, 1] × *S* ^*n*−1^) *and* **R**_*ω*_ : *L* ^2^(Ω^*n*^) → *L* ^2^([−1, 1]) *where* **R**_ω_ *f*(*s*) = **R** *f*(*s, ω*) *are linear and continuous with bounded adjoints* R* : *L* ^2^([−1, 1] × *S* ^*n*−1^) → *L* ^2^(Ω^*n*^) *and* ${\text{R}}_{\omega }^{*}$ : *L* ^2^([−1, 1]) → *L* ^2^(Ω^*n*^) *represented by*

(20) $$\begin{array}{c} {\text{R}}_{\omega }^{*}g\left(x\right)=g\left(\langle x,\omega \rangle \right),\\ {\text{R}}^{*}g\left(x\right)=\int_{S^{n-1}}g\left(\langle x,\omega \rangle ,\omega \right)\text{d}\omega \end{array}$$.

## 3. A CONNECTION BETWEEN RADON AND CONE BEAM TRANSFORM

The proof of (4) essentially relies on the duality of the pairs (**R**_*ω*_, ${\text{R}}_{\omega }^{*}$), (**D**_*a*_, ${\text{D}}_{a}^{*}$) on the one side and the fact that *δ* ^(*k*)^, where *δ* denotes Dirac's delta distribution, is homogeneous of degree −*k* −1 on the other side. To see the latter property, we take *φ* ∈ ${𝒞}_{0}^{\infty }$ (ℝ), *λ* > 0 and compute

(21) $$\begin{array}{c} \int_{\mathbb{R}}\varphi \left(s\right)\delta^{\left(k\right)}\left(\lambda s\right)\text{d}s=\lambda^{-1}\int_{\mathbb{R}}\varphi \left(\lambda^{-1}s\right)\delta^{\left(k\right)}\left(s\right)\text{d}s\\ =\lambda^{-1}{\left(-1\right)}^{k}\frac{\partial^{k}}{\partial s^{k}}{\left\{\varphi \left(\lambda^{-1}s\right)\right\}}_{{|}_{s=0}}\\ =\lambda^{-k-1}{\left(-1\right)}^{k}\varphi^{\left(k\right)}\left(0\right)\\ =\int_{\mathbb{R}}\varphi \left(s\right)\lambda^{-k-1}\delta^{\left(k\right)}\left(s\right)\text{d}s. \end{array}$$

For a tensor field **f** ∈ *L* ^2^(Ω*^n^, 𝒮^m^*) and *a* ∈ Γ, we furthermore define

(22) $$\begin{array}{c} fa\left(x\right)=\langle \text{f}\left(x\right),{\left|x-a\right|}^{-m}{\left(x-a\right)}^{m}\rangle \\ =f_{i_{1}\cdots i_{m}}\left(x\right){\left|x-a\right|}^{-m}{\left(x-a\right)}^{i_{1}}\cdots {\left(x-a\right)}^{i_{m}},1\leq i_{j}\leq n,j=1,\ldots ,m. \end{array}$$

Using the Cauchy-Schwartz inequality, we easily get

(23) $$\int_{\Omega^{n}}{\left|f_{a}\left(x\right)\right|}^{2}\text{d}x\leq \int_{\Omega^{n}}{\left\|\text{f}\left(x\right)\right\|}^{2}\text{d}x.$$

Thus, *f_a_* ∈ *L* ^2^(Ω*^n^*), when **f** ∈ *L* ^2^(Ω*^n^, 𝒮^m^*).

We are now able to state the main result of this paper.

**Theorem 2.**

*Let n* ≥ 2 *and* **f** ∈ ${𝒞}_{0}^{\left(n-2\right)}$ (Ω*^n^, 𝒮^m^*). Then

(24) $$\frac{\partial^{\left(n-2\right)}}{\partial s^{\left(n-2\right)}}\text{R}fa\left(\omega ,s=\langle a,\omega \rangle \right)={\left(-1\right)}^{\left(n-2\right)}\int_{S^{n-1}}\text{Df}\left(a,\theta \right)\delta^{\left(n-2\right)}\left(\langle \omega ,\theta \rangle \right)\text{d}\theta$$,

*where a* ∈ Γ, *ω* ∈ *S* ^*n*−1^.

**Proof.**

We follow the proof of Grangeat's classical formula as outlined in Natterer and Wübbeling [11, Section 2.3]. For *ψ* ∈ *L* ^2^([−1, +1]), we have from lemma 2 that

(25) $$\begin{array}{c} \int_{-1}^{+1}{\text{R}}_{\omega }f_{a}\left(s\right)\psi \left(s\right)\text{d}s=\int_{\Omega^{n}}f_{a}\left(x\right)\psi \left(\langle x,\omega \rangle \right)\text{d}x\\ =\int_{\Omega^{n}}\langle \text{f}\left(x\right),{\left|x-a\right|}^{-m}{\left(x-a\right)}^{m}\rangle \psi \left(\langle x,\omega \rangle \right)\text{d}x. \end{array}$$

Using (15), we obtain in the same way for *h* ∈ *L* ^2^(*S* ^*n*−1^),

(26) $$\int_{S^{n-1}}{\text{D}}_{a}\text{f}\left(\theta \right)h\left(\theta \right)\text{d}\theta =\int_{\Omega^{n}}\langle \text{f}\left(x\right),{\left|x-a\right|}^{1-n-m}{\left(x-a\right)}^{m}\rangle h\left(\frac{x-a}{\left|x-a\right|}\right)\text{d}x.$$

Assertion (24) is then proved when setting *h*(*θ*) = *δ* ^(*n*−2)^(〈*θ, ω*〉), *ψ*(*s*) = *δ* ^(*n*−2)^(*s* − 〈*a, ω*〉) and taking into account that *δ* ^(*n*−2)^ is homogeneous of degree 1 − *n*.

**Remark 2.**

Obviously, *δ* ^(*n*−2)^ is not in *L* ^2^([−1, +1]). But since *δ* ^(*n*−2)^ ∈ (*𝒞* ^(*n*−2)^([−1, +1]))′ and the cone beam transform **D** **f**(*a, y*) can be extended homogeneously to ℝ*^n^* with respect to the second variable for any *m* according to *m* = 1 (see [11, Section 2.3]), the integrals in the proof of Theorem 2 are well defined by the smoothness requirement for **f**. The expression on the right-hand side of (24) is to be understood as

(27) $${\left(-1\right)}^{\left(n-2\right)}\int_{S^{n-1}}\text{Df}\left(a,\theta \right)\delta^{\left(n-2\right)}\left(\langle \omega ,\theta \rangle \right)\text{d}\theta =\int_{S^{n-1}\cap \omega^{⊥}}\langle d^{\left(n-2\right)}\text{Df}\left(a,y=\theta \right),\omega^{\left(n-2\right)}\rangle \text{d}\mathrm{\theta ,}$$

where *d^m^ = d* ⊗ ⋯ ⊗ *d* means the *m*-fold inner derivative with respect to the second variable in **D** **f**(*a, y*). We have that *d* ^1^ = ∇ is the gradient, *d* ^2^ is the Hessian.

If *n* = 3, *m* = 0, (24) is just the classical formula of Grangeat (3). For *m* = 1, we get an extension of Grangeat's formula to vector fields, where

(28) $$f_{a}\left(x\right)=\langle \text{f}\left(x\right),{\left|x-a\right|}^{-1}\left(x-a\right)\rangle .$$

The benefits of formula (24) can barely be anticipated. Let us consider the scalar case, that is, *m* = 0. If there exists to each *s* ∈ [−1, 1] a source point *a* ∈ Γ such that 〈*a, ω*〉 = *s*, then the derivative $\partial_{s}^{\left(n-2\right)}$ **R** *f*(*ω, s*) can be obtained for arbitrary *ω* ∈ *S* ^*n*−1^, *s* ∈ [−1, 1] by integrating a corresponding derivative of the data **D** *f *(*a, θ*) over the manifold *S* ^*n*−1^ ∩ *ω* ^⊥^. This condition is well known as *Tuy's condition* (see, e.g., [10, Section VI.5]) and means that every hyperplane passing through Ω*^n^* has to intersect the source curve Γ in at least one point. The situation changes decisively for *m* > 0 since the projections *f_a_* depend on the source point *a*. Even if we found to every *s* a source *a* satisfying 〈*a, ω*〉 = *s*, this would not help since the object function *f_a_* of **R** changes with *a*. Thus applying formula (24) would give us **R** *f_a_* (*ω, s*) for a *single s*, namely, *s* = 〈*a, ω*〉. Tuy's condition is not sufficient for *m* > 0. Moreover, we have to take into account that there is a nontrivial null space for *m* > 0 anyway. To see this, we note that **D** **f** = 0 if **f** is a *potential field*, that means **f** = *d* **p** for **p** ∈ $H_{0}^{1}$(Ω*^n^, 𝒮* ^*m*−1^). We refer to the book of Sharafutdinov [9] for a characterization of the null space of **D**. Denisjuk [12] suggested a generalization of Tuy's condition for higher order tensor fields. He obtained similar formulas as (24) and showed that every plane through Ω*^n^* has to intersect Γ at least *m* − 1 times.

If it is possible to compute *f_a_* with the help of formula (24), the curve Γ additionally has to satisfy the requirement that **f**(*x*) can be computed from the projections

(29) $$\langle \text{f}\left(x\right),{\left|x-a\right|}^{-m}{\left(x-a\right)}^{m}\rangle$$.

This is possible, if the curve Γ fulfills the condition, that for each *x* ∈ Ω*^n^* there exist dim(*𝒮^m^*) = *n^m^* source points *a*_1_,…,*a_n_^m^* such that the tensors |*x − a_i_*|*^−m^* (*x − a_i_*)*^m^* are linearly independent for fixed *x* and 1 ≤ *i* ≤ *n^m^*. The tensor field **f**(*x*) can then be recovered from the projections (29). In case of three-dimensional vector fields (*n* = 3, *m* = 1), we need three linearly independent vectors *x − a_i_* to each *x*. Hence, this condition is not fulfilled when, for example, Γ = {*a* ∈ ℝ^3^ : |*a − a*_0_| = *r*, *a*_3_ = 0} is a single circle since we find no such vectors for *x* in {|*x − a*_0_| < 1, *x*_3_ = 0}.

Formula (24) could be used to calculate reconstruction kernels for **D**, that is we could try to solve

(30) $${\text{D}}^{*}\nu_{i_{1}\cdots i_{m}}^{\gamma }\left(x\right)={\text{E}}_{i_{1}\cdots i_{m}}^{\gamma }\left(x,\cdot \right)$$

using that relation to the Radon transform, where ${\text{E}}_{i_{1}\cdots i_{m}}^{\gamma }$ (*x, y*) ≈ *δ*(*x − y*)$dx^{i_{1}}$ ⊗ ⋯ ⊗ $dx^{i_{m}}$ for small *γ* > 0 is an approximation to the delta distribution. Reconstruction kernels are necessary to cope the problem of tensor tomography with the *method of approximate inverse*; see, for example, Louis [13], Schuster [3], Rieder and Schuster [14]. It is clear that

(31) $$\text{Df}\left(a,\omega \right)\omega +\alpha_{1}\left(a,\omega ,\omega_{1}\right)\omega_{1}+\alpha_{2}\left(a,\omega ,\omega_{2}\right)\omega_{2}=\int_{0}^{\infty }\text{f}\left(a+t\omega \right)\text{d}t$$

for certain coefficients *α*_1_, *α*_2_, where {*ω, ω*_1_, *ω*_2_} forms an orthonormal basis of ℝ^3^. Unfortunately, *α*_1_, *α*_2_, are unknown. An idea to apply the method of approximate inverse to **D** might be to approximate

(32) $$\text{Df}\left(a,\omega \right)\omega \approx \int_{0}^{\infty }\text{f}\left(a+t\omega \right)\text{d}t$$,

and to use methods for 3D cone beam CT to solve the problem. If *ν^γ^*(*x*) denotes a reconstruction kernel for **D** in case *m* = 0, then $\nu_{i}^{\gamma }$ (*x*) := *ν^γ^* (*x*) · *e_i_* represents a reconstruction kernel for the right-hand side of (32). This approach is subject of current research. Hence, relation (24) might be of large interest in the area of tensor tomography problems.
